# Histiocytic Necrotizing Lymphadenitis Mimicking Acute Appendicitis in a Child: A Case Report

**DOI:** 10.3389/fped.2021.682738

**Published:** 2021-09-17

**Authors:** Chun-Zhen Hua, Yu-Kai Chen, Shun-Zhi Chen, Wei-Zhong Gu, Qiang Shu

**Affiliations:** ^1^Department of Infectious Diseases, Children's Hospital, Zhejiang University School of Medicine, National Clinical Research Center for Child Health, Hangzhou, China; ^2^Department of General Surgery, The Second Affiliated Hospital, Zhejiang University School of Medicine, Hangzhou, China; ^3^Department of Pediatric Surgery, Shaoxing People's Hospital, Shaoxing, China; ^4^Department of Pathology, The Children's Hospital, Zhejiang University School of Medicine, National Clinical Research Center for Child Health, Hangzhou, China; ^5^Department of Pediatric Surgery, The Children's Hospital, Zhejiang University School of Medicine, National Clinical Research Center for Child Health, Hangzhou, China

**Keywords:** case report, acute appendicitis, Kikuchi-Fujimoto disease, histiocytic necrotizing lymphadenitis, child

## Abstract

**Background:** Histiocytic necrotizing lymphadenitis, also known as Kikuchi-Fujimoto disease (KFD), is a self-limiting inflammatory disease with low incidence and high misdiagnosis rate in children. Furthermore, cases where the clinical presentation resembles acute appendicitis are very rare.

**Case Presentation:** A 14-year-old boy was misdiagnosed as acute appendicitis and received operative treatment at his early visit. He suffered from abdominal pain, vomiting, diarrhea, fever, and lymphadenitis at the ileocecal junction, which were found by B-ultrasonography examination and surgery. Lymphadenectomy, as well as appendectomy, was performed, and KFD was identified by pathological examination. The patient was transferred to our hospital for further therapy because of recurrent fever and abdominal pain after the appendectomy. His temperature became normal after methylprednisolone was administered, and no recurrence was observed till now during follow-up.

**Conclusions:** Necrotizing lymphadenitis involving mesenteric lymph nodes may cause acute-appendicitis-like symptom; KFD should be a diagnostic consideration for mesenteric lymphadenitis.

## Introduction

Histiocytic necrotizing lymphadenitis, also known as Kikuchi-Fujimoto disease (KFD), is a self-limiting inflammatory disease with nonneoplastic lymphadenectasis. It occurs more frequently in older children, with major clinical manifestations of cervical lymphadenopathy and long-term fever (1–4). The laboratory findings could be leukopenia and/or neutropenia, sometimes accompanied by increased levels of alanine aminotransferase (ALT) and/or aspartate aminotransferase (AST) in serum (1–5). As a disease with low incidence, KFD is easily misdiagnosed as other diseases, such as lymphoma, lymph node tuberculosis, infectious mononucleosis, and Kawasaki disease (6–9). However, initial symptom presenting as acute abdomen was rarely reported. Here, we reported a patient diagnosed initially with acute appendicitis who had necrotizing lymphadenitis in the ileocecal junction identified by surgical finding and pathological examination.

## Case Presentation

**Figure 1:**
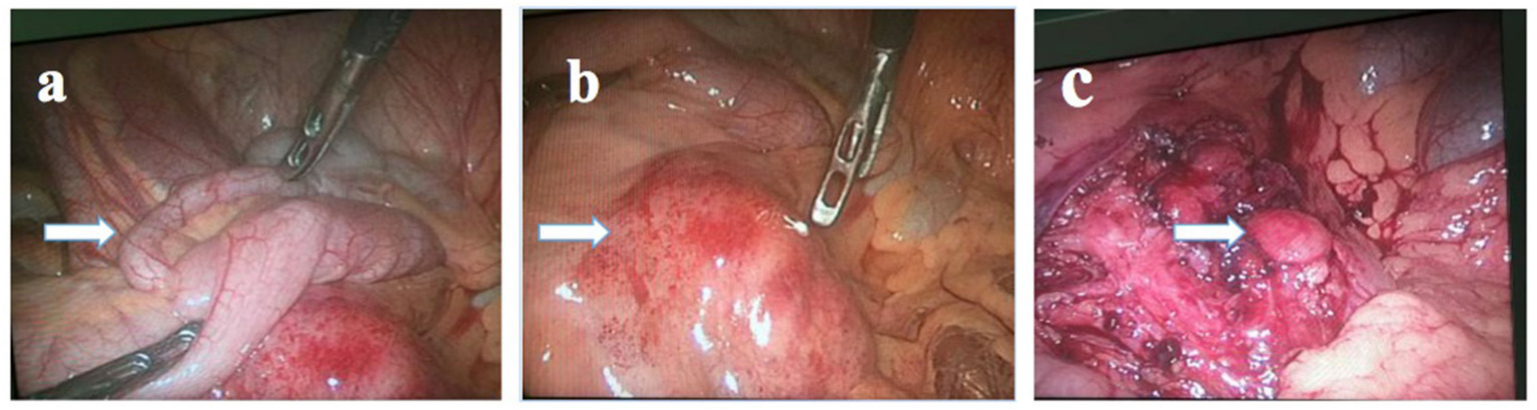
Preoperative and postoperative visual field examinations of the patient. **(a)** The arrowed tissue was the appendix. **(b)** The arrowed tissue was an enlarged mesenteric lymph node in the ileocecal region. **(c)** The arrowed tissue was another enlarged lymph node which was not removed.

**Figure 2:**
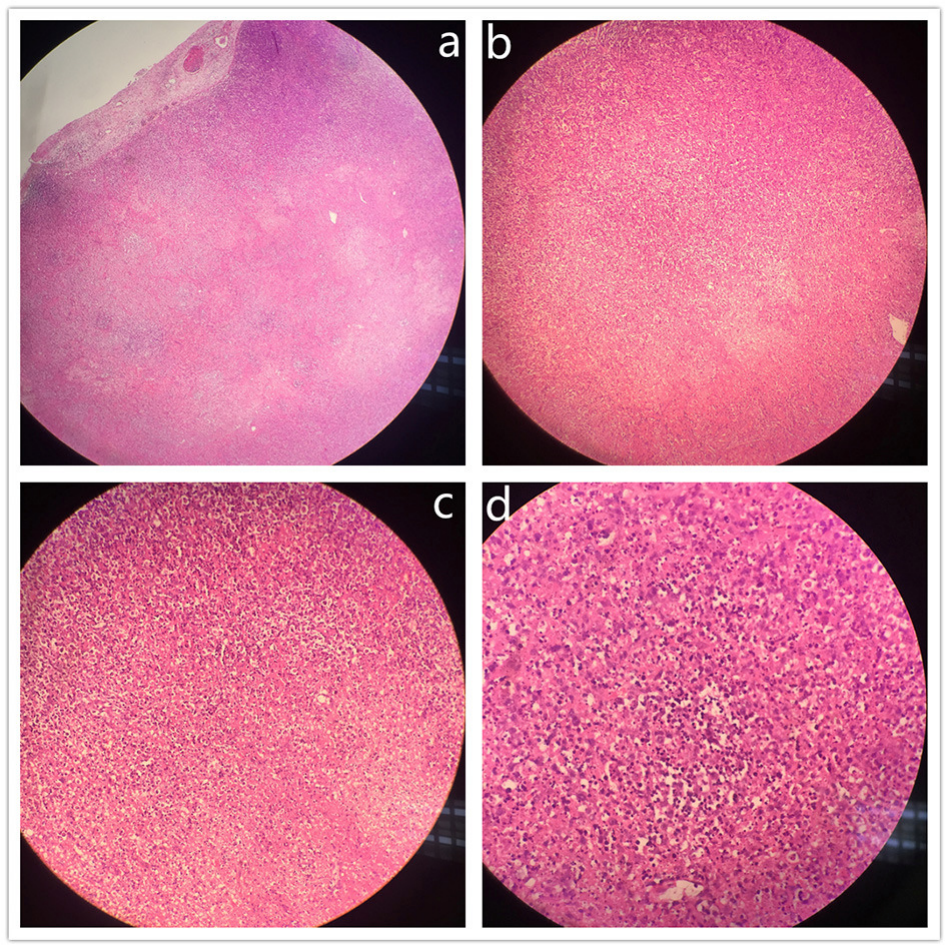
Histopathological findings of lymph node biopsy. Lymph node sections showed partially preserved lymphoid architecture with lymphoid follicles and paracortical coagulative necrosis with karyorrhectic debris and apoptosis, absence of neutrophils, and presence of histiocytes and lymphoid cells. (H&E stain; **(a)** original magnification ×40; **(b)**, original magnification ×100; **(c)**, original magnification ×200; **(d)**, original magnification ×400).

A 14-year-old boy presented to the Children's Hospital, Zhejiang University School of Medicine, with main complaints of recurrent abdominal pain accompanied by fever for 14 days. He had middle and upper abdominal pain on day 1 of presentation, accompanied by vomiting and diarrhea. He was diagnosed with acute gastroenteritis at a local clinic, and the symptoms relieved after being administered with intravenous cefuroxime and anisodamine. On day 2 of presentation, his abdominal pain worsened and localized at the right lower abdomen. He also had a low-grade fever, with a temperature of 38.0 °C. Thus, he went to Shaoxing People's Hospital, and physical examination showed obvious pressing pain at McBurney's point at the right lower abdomen. B-ultrasound examination revealed appendicitis (diameter 6 mm) and several lymph node echoes in the right lower abdomen, the largest size of which was 21 × 10 mm. The surgeon considered it was acute appendicitis, and an emergency laparoscopic operation was performed. During the operation, about 10 ml of light yellow transparent liquid was sucked from the ileocecal junction. The normal appendix and swollen lymph nodes in dark red were found in the operative field (Figure 1). Lymphadenectomy, as well as appendectomy, was performed for pathological examination. After the operation, intravenous cefotiam and metronidazole were prescribed empirically, and symptom of abdominal pain was relieved. Since day 3 postoperation (day 5 of presentation), his temperature decreased to near normal level. The pathological examination indicated almost normal appendix and necrotizing lymphadenitis (Figure 2). The patient was discharged from Shaoxing People's Hospital on day 6 postoperation (day 8 of presentation) with body temperature at 37.7 °C.

After discharge, the boy had low-grade fever accompanied by mild abdominal pain occasionally. Since day 10 of presentation, his body temperature rose to as high as 39.4 °C. Intravenous ceftriaxone for 4 days was prescribed in a local clinic, and his symptoms did not improve at all.

As a result, the boy was moved to the Children's Hospital, Zhejiang University School of Medicine. During the course of his illness, he had poor appetite and sleep. He had no past medical history, and his parents denied familial inherited disease. Physical examination on admission finding includes the following: temperature of 38.6°C, heart rate at 100 number/minute (n/min), respiration at 24 n/min, blood pressure at 117/90 mmHg, weight of 53 kg, and height at 170 cm. Enlarged lymph nodes without tenderness were found in the cervical, axillary, and inguinal area. The size of the largest one was 15 × 10 mm. Heart and lung exams were normal. No abdominal tenderness was evoked during abdominal examination. The liver and spleen were not palpable. The diagnoses were KFD and postoperative appendectomy. After admission, the results of the PPD test, mycoplasma antibodies detection, tuberculosis T-SPOT test, and autoantibody test were all negative. The level of procalcitonin in serum was normal. On the fourth day after admission (on day 17 of presentation), the patient agreed to receive intravenous methylprednisolone, and his body temperature dropped to normal at the same day. After being treated with methylprednisolone for 5 days (intravenous for 3 days, 1 mg/(kg/d), twice a day; oral for 3 days, 0.5 mg/(kg/d), twice a day), he was discharged without any symptoms. He completed therapy with methylprednisolone according to the doctor's advice, including oral methylprednisolone 6 mg twice a day for 3 days, 6 mg once a day for 3 days, and 2 mg once a day for 7 days. He was followed up for 2 years after discharge, and no similar symptoms or abnormal laboratory findings recurred. The laboratory findings of the patient during the course of illness are shown in Table 1.

**Table 1:** Laboratory findings during the course of illness in the patient.

**Days of presentation**	**WBC × 10^**9**^/L**	**N × 10^**9**^/L**	**CRP (mg/L)**	**ALT (U/L)**	**AST (U/L)**
Day 2	5.46	3.07	13.25	9.7	17
Day 7	4.87	2.49	14.72	8.2	15.9
Day 13	4.3	2.15	6.26	–	–
Day 15	3.86	1.92	1.65	19	32
Day 22	4.92	2.11	2.22	93	83
Day 29	12.63	6.83	0.14	47.1	16.8

*WBC, white blood cell; N, neutrophils; CRP, C-reaction protein; ALT, alanine aminotransferase; AST, aspartate aminotransferase*.

## Discussion and Conclusions

KFD is a disease with unclear etiology and pathogenesis (3, 10). It occurs more frequently in Asians (10–12), and the clinical and pathological features are similar to that in virus infectious disease. EB virus, herpes simplex virus, varicella-zoster virus, parainfluenza virus, and rubella virus might be associated with KFD. However, there was insufficient evidence to ascribe KFD to a particular virus. KFD might have some relationship to various autoimmune diseases (13). However, serologic markers for autoimmune disease, such as rheumatoid factor, antinuclear antibodies, and antidouble-strand DNA antibodies, are negative in most patients (12–14). This boy was an older child, with no obvious disease process associated with KFD, such as: (1) EB virus infection, tuberculosis, or mycoplasma infection were not identified; (2) all of the tested autoimmune antibodies were negative; (3) the result of blood routine showed normal or nearly normal leukocyte count and neutrophil count; (4) hepatic function damage had been identified by increased ALT and AST in serum; and (5) antibiotic therapy did not improve the symptoms at all. These mentioned features were all in accordance with the manifestation of KFD. The boy had illness onset as appendicitis-like pain in the right lower quadrant of the abdomen, as well as vomiting, diarrhea, and fever. Physical examination showed apparent tenderness at McBurney's point. B-ultrasound examination found the echo of the appendix and swollen lymph nodes, which supported for indications of emergency surgery. During the operation, appendix and node-like masses in dark red were excised. It is possible that the swollen lymph nodes might have stimulated the intestinal tract and caused gastrointestinal symptoms. The diagnosis of KFD mainly depends on the pathological examination of the lymph node. Similarly, cervical lymphadenectasis was found when the patient was hospitalized in our hospital, and the re-examination of the original pathological sections led to the pathological diagnosis of KFD too. Steroid was prescribed for therapy, and the boy's temperature fell to normal soon. The treatment process went smoothly, and there has been no recurrence so far.

It is challenging to distinguish KFD from other diseases at the early stage because of nonspecific clinical manifestation. As a result, the rate of misdiagnosis in patients with KFD was high at their first visit (6, 7). Lymph node biopsy is the only way to diagnose this disease. Usually, lymphadenectasis occurs in the neck (1, 3, 15), from which it is more convenient for excision biopsy. The main lymphadenopathy in this patient was mesenteric lymph node. If there was no manifestation of an acute abdominal pain, the affected lymph node might not be excised through surgery in time, and the diagnosis process of KFD might be more tortuous. Three KFD cases involving mesenteric lymph nodes and causing acute-appendicitis-like symptoms were reported, and the patients were adults who underwent appendectomy with excision of an enlarged mesenteric lymph node and were diagnosed as KFD based on histopathological findings (16–18). The pathological features showed lymph node has architecture with partial follicular hyperplasia. The paracortex is expanded with patchy, well-circumscribed areas of necrosis, scattered with crescentic histiocytes. Among the histiocytes, small lymphocytes, activated T cells, and some plasma cells are scattered, but neutrophils and eosinophils were absent. At the edge of necrosis areas where abundant nuclear debris and histiocytes gathered, thrombosis could be seen as well (8).

Basically, KFD is a self-limiting disease that mainly depends on symptomatic treatment. The use of steroids could shorten the clinical course, especially recommended for those recurrent patients or those with severe clinical symptoms (19–21). Hydroxychloroquine might be an alternative for steroids because of its effectiveness and safety (22). It was reported that the body temperature might drop to normal after excision biopsy in some patients (3, 4). Selvanathan et al. reported in his study that symptoms relieved in 25.5% of patients the next day after biopsy and in 43.9% of patients 1 week after biopsy. The mechanism of improvement of symptoms might be related to the complete excision of the lesion tissues. In this patient, swollen lymph nodes were not excised completely by operation. The residual necrotic lymph node might have caused fever relapse in this patient. KFD is prone to relapse, and the recurrence rate is up to 42.4% in children (21, 23–25). It might also develop into other autoimmune diseases, such as SLE (4, 11, 25, 26). The follow-up time for this patient is not long enough to observe the long-term outcome, and the patient should accept further follow-up in the coming decades.

In summary, KFD is a rare benign self-limiting disease, and cervical lymphadenopathy and fever are the most common onset. It lacks specific clinical characteristics and is easy to be misdiagnosed as lymphoma, lymph node tuberculosis, and other diseases. In this patient, the necrotic lymph nodes were in the ileocecal junction in the abdominal cavity and caused acute-abdomen-like manifestation, indicating that when analyzing lymphadenopathy, KFD should be considered regardless of the parts where lymphadenopathy occurred. If necessary, lymph node biopsy is advised to avoid misdiagnosis and inappropriate treatment.

## Data Availability Statement

Within 6 months of this publication, anonymised individual participant data will be available (from the corresponding author) for research proposals approved by an independent review committee. Proposals should be submitted to shuqiang@zju.edu.cn. A data access agreement will be required.

## Ethics Statement

The studies involving human participants were reviewed and approved by the Institutional Ethics Board of the Children's Hospital, Zhejiang University School of Medicine (2020-IRB-137). Written informed consent to participate in this study was provided by the participants' legal guardian. Written informed consent was obtained from the individual(s), and minor(s)' legal guardian, for the publication of any potentially identifiable images or data included in this article.

## Author Contributions

CZH treated the patient during his hospitalization, conceptualized and designed the report, drafted the initial manuscript, and reviewed and revised the manuscript. YKC collected the clinical and laboratory data, drafted the initial manuscript, and reviewed the manuscript. SZC operated lymphadenectomy and appendectomy, collected the clinical data, carried out the initial analyses, and reviewed the manuscript. WZG diagnosed the patient pathologically, checked the data, and reviewed and revised the manuscript. QS conceptualized and designed the report, coordinated data collection, and critically reviewed the manuscript for important intellectual content. All authors approved the final manuscript as submitted and agree to be accountable for all aspects of the work.

## Conflict of Interest

The authors declare that the research was conducted in the absence of any commercial or financial relationships that could be construed as a potential conflict of interest.

## Publisher's Note

All claims expressed in this article are solely those of the authors and do not necessarily represent those of their affiliated organizations, or those of the publisher, the editors and the reviewers. Any product that may be evaluated in this article, or claim that may be made by its manufacturer, is not guaranteed or endorsed by the publisher.
